# Primary and memory immune responses against rough *Brucella canis* are less robust compared to smooth *B. abortus* and *B. melitensis* following intratracheal infection in mice

**DOI:** 10.3389/fimmu.2022.959328

**Published:** 2022-08-11

**Authors:** Lauren W. Stranahan, Daniel G. Garcia-Gonzalez, Martha E. Hensel, Angela M. Arenas-Gamboa

**Affiliations:** Department of Veterinary Pathobiology, College of Veterinary Medicine, Texas A&M University, College Station, TX, United States

**Keywords:** *Brucella canis*, intratracheal, brucellosis, vaccine, polyfunctional, immune response, lipopolysaccharide

## Abstract

*Brucella canis* is the cause of canine brucellosis, a globally distributed, zoonotic pathogen which primarily causes disease in dogs. *B. canis* is unique amongst the zoonotic *Brucella* spp. with its rough lipopolysaccharide, a trait typically associated with attenuation in gram-negative bacteria. Unfortunately, no vaccine is available against *B. canis*, and vaccine development is hampered by a limited understanding of the immune response required to combat it and the course of infection following a physiologically relevant, mucosal route of inoculation. To address these concerns and analyze the impact of the rough phenotype on the immune response, we infected mice intratracheally with rough *B. canis* or smooth *B. melitensis* or *B. abortus*. Bacterial colonization and histologic lesions were assessed in systemic target organs as well as locally in the lungs and draining mediastinal lymph node. Mice were also reinfected with *Brucella* following antibiotic treatment and cytokine production by T lymphocytes in the lung and spleen was assessed by flow cytometry to investigate the memory immune response. Despite its rough phenotype, *B. canis* established a persistent infection at the same level of colonization as the smooth strains. However, *B. canis* induced significantly less granulomatous inflammation in the spleen as well as a lack of bronchial-associated lymphoid tissue (BALT) hyperplasia in the lungs. These differences coincided with increased IL-10 and decreased IFN-γ in the spleen of *B. canis*-infected mice. Previous exposure to all *Brucella* strains provided protection against colonization following secondary challenge, although induction of IFN-γ by T lymphocytes was seen only in the lungs during *B. canis* infection while the smooth strains induced this cytokine in the spleen as well. Neither *Brucella* strain induced significant polyfunctional T lymphocytes, a potential immunomodulatory mechanism that appears to be independent of lipopolysaccharide phenotype.

## Introduction

Brucellosis, caused by the gram-negative, facultatively intracellular bacterium *Brucella*, is one of the most common zoonotic diseases globally, with widespread and serious public health consequences (1, 2). Human infections are usually associated with *B. melitensis, B. abortus*, and *B. suis* harbored predominantly by small ruminants, cattle, and pigs, respectively (3, 4). However, humans may also become infected with *Brucella canis*, a pathogen which primarily causes reproductive disease in dogs (5, 6). In stark contrast to the other zoonotic *Brucella* spp., *B. canis* possesses a rough lipopolysaccharide (LPS) which lacks the terminal O-polysaccharide (O-PS), a feature which is typically associated with attenuation in gram-negative bacteria (7, 8). While thought to be less virulent for humans, manifestation of disease following *B. canis* infection can occasionally be severe, including osteomyelitis and endocarditis (9, 10). Cases of canine brucellosis have been reported with increasing frequency worldwide, corresponding to rising reports of infection in humans (6, 10–14). Due to numerous factors, including difficulties in diagnosis and lack of specificity of clinical signs, vaccination represents the only rational strategy to protect human and animal populations from brucellosis. Unfortunately, while vaccines are commercially available to protect livestock from smooth *Brucella* spp., no vaccine exists to defend against *B. canis* (15).

Numerous obstacles exist in developing a vaccine against *B. canis*, including lack of characterization of infection in the mouse model following mucosal exposure and a dearth of knowledge regarding immune correlates of a protective memory response. Mice have been heavily utilized in the longstanding quest to develop new vaccines against brucellosis, both in testing the safety and efficacy of candidates and in dissecting the associated protective immune response (16). Recent work has characterized *B. canis* infection in mice following the classic intraperitoneal (i.p.) route of inoculation, suggesting that *B. canis* is less virulent in mice than *B. abortus* and *B. melitensis*, both in terms of the dose required to cause systemic colonization and the severity of the inflammatory response in target organs (17, 18). In recent years, increasing efforts have been made to evaluate the pathogenesis of brucellosis and identify correlates of immune protection in mice using mucosal inoculation rather than the traditional i.p. approach. Brucellosis in both humans and animals is most frequently acquired *via* oral or respiratory exposure and there is compelling evidence that the immune response required to defend against pathogens invading across mucosal surfaces differs from that required against parenteral infection (19–21). While several studies have evaluated the protective immune response against mucosal infection by smooth *Brucella* spp., this area remains completely unexplored for *B. canis*, representing a significant impediment towards vaccine development (22).

An additional barrier to develop a *B. canis* vaccine is an incomplete understanding of the components of protective memory immune response against infection. The critical importance of a Th1 response with central involvement of the MyD88/IL-12 signaling pathway and IFN-γ produced by CD4+ and/or CD8+ T lymphocytes against smooth *Brucella* spp. is well established (23–25). Even for the less studied *B. canis*, superior protection against challenge has been correlated with increased levels of IFN-γ in mice (26, 27). Assessment of the memory response to challenge, rather than the initial response to vaccination, is critical to understanding the components of a vaccine that would allow it to be protective. A handful of recent studies have begun to elucidate what makes up an effective memory response against smooth *Brucella* spp. using secondary infection as a model, a strategy which has also been employed for other bacterial pathogens, including *Listeria monocytogenes* (20, 21, 28, 29). Unfortunately, such investigations have not yet extended to *B. canis*, resulting in further confusion regarding rational vaccine design against this bacterium.

In this study, we extended our previous work with *B. canis* in mice by characterizing infection using a more physiologically relevant, intratracheal route of inoculation and used this model to make initial efforts into evaluating the memory immune response. Our study reveals that *B. canis* induces both a less robust primary inflammatory and secondary/memory immune response than *B. abortus* or *B. melitensis*, indicating key differences in the interaction of this rough pathogen with the immune system in comparison to its smooth counterparts.

## Materials and methods

### Ethics statement

Animal experiments were conducted in an approved facility in strict accordance with all university and federal regulations. Mouse experimental procedures (protocol: 2018-0046, 2021-0038) were reviewed and approved by the Texas A&M University Laboratory Animal Care and Use Committee (IACUC). Texas A&M University is accredited by the Association for the Assessment and Accreditation of Laboratory Animal Care, International (AAALAC).

### Animals

Female C57BL/6J mice (9-12 weeks old) were obtained from the Texas A&M Institute for Genomic Medicine (TIGM) and housed in microisolator caging in biosafety level 2 and 3 facilities at Texas A&M University College of Veterinary Medicine. C57BL/6J mice were selected over BALB/c as this strain was previously used to characterize *B. canis* infection in mice following i.p. inoculation. While clearance of infection is achieved earlier in C57BL/6J mice, initial organ colonization and replication profiles are similar to BALB/c. All mice were acclimated to the facility for 5 days prior to vaccination or infection and were maintained on a 12-hour—12-hour light-dark cycle with *ad libitum* access to food and filtered water. Mice were monitored daily for signs of pain or distress according to the guidelines of the Animal Research Advisory Committee published by the National Institutes of Health.

### Bacterial strains

*B. abortus* 2308 was obtained from the National Animal Disease Center in Ames, Iowa while *B. canis* RM6/66 was acquired from ATCC. *B. melitensis* 16M was obtained originally from ATCC and reisolated by this lab from an aborted goat fetus (30). Mutant strain *B. canis* RM6/66 *ΔvjbR* as generated by this lab in a previous study (31). Bacterial stocks were stored at -80°C in 25% glycerol and were routinely grown on tryptic soy agar (TSA) plates or in standard tryptic soy broth (TSB). Bacteria were harvested from plates grown for 3-4 days using phosphate-buffered saline (PBS), pH 7.2 (Gibco) and adjusted to a final concentration of either 10^7^ or 10^9^ CFU per 25-30 µL dose using a Klett colorimeter meter reading against a standard curve. Viable counts were retrospectively confirmed by serial dilution and plating onto TSA plates. For cell culture infection, bacteria were harvested from plates and grown in TSB at 37°C and 200 rpm for 16-18 h.

### Mice infection or vaccination

For all experiments, mice were randomly divided into groups (n= 5-7). To characterize the kinetics of organ colonization, mice were infected intratracheally with a 25 µL dose containing 10^7^ CFU of *B. abortus* 2308, *B. melitensis* 16M, or *B. canis* RM6/66, or 10^9^ CFU of *B. canis* RM6/66, as previously described (32). Briefly, mice were anesthetized *via* intraperitoneal (i.p.) injection of 100 mg/kg ketamine and 10 mg/kg xylazine diluted in sterile PBS. Mice were then placed in dorsal recumbency on a plexiglass support at a 45° angle and the larynx was visualized using a small animal laryngoscope (Model LS-2-M, Penn Century Inc) with the aid of a magnifying glass. The inoculum was administered using the PennCentury™ MicroSprayer IA-1C device placed distal to the larynx (Penn Century Inc).

In other experiments, mice were vaccinated with 10^9^ CFU of the vaccine candidate, *B. canis ΔvjbR* either subcutaneously in 100 µL PBS or intranasally in 30 µL PBS. For intranasal vaccination, mice were anesthetized with ketamine and xylazine and the inoculum was administered evenly to each nostril using a long-reach 200 µL pipette tip. The vaccination dose was established in a previous study (31). Mice were then challenged at 6-weeks post-vaccination and euthanized 2-weeks post-challenge to evaluate protective efficacy.

### Antibiotic treatment

In some experiments, antibiotics were administered to mice beginning at 4-weeks post-infection with *Brucella* spp. and lasting for 3 weeks. Mice were injected i.p. daily with 150 mg/kg streptomycin and 20 mg/kg rifampin dissolved in endotoxin-free PBS and filter-sterilized. Mice were then rested for 2 weeks to allow for clearance of antibiotics. To ensure that antibiotic treatment resulted in clearance of bacteria, 2-3 mice from each group were sacrificed 1 week prior to challenge (9-weeks post-infection) and liver, spleen, lung, and mediastinal lymph node were evaluated for bacterial colonization.

### Kinetics of organ colonization

Mice were sacrificed by CO_2_ asphyxiation and cervical dislocation at 1-, 2-, 3-, 4-, 6-, and 9-weeks post-infection. At each time point, samples of liver, spleen, uterus, lung, mediastinal lymph node, and kidney were collected into 1 mL PBS, homogenized using an Omni TH homogenizer (Omni International), serially diluted, plated on Farrell’s medium (TSA plus *Brucella* Oxoid supplement, equine serum, and 50% dextrose), and incubated at 37°C. Bacterial colonies were enumerated after 72 h to quantify tissue colonization and levels of infection were expressed as the log_10_ number of CFU per gram of tissue.

### Histology and immunohistochemistry

Samples of spleen, liver, lung, uterus, cervical lymph node, mesenteric lymph node, heart, and kidney were collected at necropsy and fixed in 10% neutral buffered formalin for a minimum of 48 h. Tissues were routinely processed and embedded, sectioned at 4 µm, and stained with hematoxylin and eosin. Sections from the spleen and liver at 2-weeks post-infection were analyzed for severity of granulomatous inflammation by a board-certified veterinary anatomic pathologist (LWS) using QuPath Bioimage analysis v 0.1.2 (33). Foci of granulomatous inflammation were annotated and % of total tissue area affected was calculated.

Unstained sections from the aforementioned organs were adhered to positively charged glass slides for immunohistochemistry (**Figure 2E**, **Supplemental Figure 3E**). Slides were deparaffinized and rehydrated through a series of xylene and ethanol steps before antigen retrieval using 1:10 EMS Solution A (Electron Microscopy Services) in a 2100 Antigen Retriever (Aptum Biologics Ltd) according to the manufacturer’s instructions. Endogenous peroxidases were blocked by a 10 min incubation with Bloxall Blocking Solution (Vector Laboratories) followed by a 20 min block of nonspecific binding in diluted normal goat serum (Vector Laboratories). Slides were incubated overnight at 4°C with a rabbit polyclonal anti-*Brucella* antibody (Bioss) at 1:400. Vectastain ABC and Betazoid DAB chromagen kits (Biocare Medical) were used according to the manufacturer’s instructions. Following each step, slides were washed in PBS + 0.05% Tween-20 (PBST) for 5 min. Slides were counterstained with Gill’s hematoxylin, dehydrated, and examined using light microscopy. Negative controls included section from uninfected animals and sections without exposure to primary antibody.

### Humoral immune response

Blood was collected from mice at each euthanasia time point and at biweekly intervals from vaccinated mice *via* the lateral tail vein and stored at 4°C for 24 h. Following centrifugation at 3000 rpm for 5 min, serum was sterilized using Corning^R^ Costar^R^ Spin-X^R^ plastic centrifuge tube filters at 10,000 g for 2 min. Anti-*Brucella* specific immunoglobulin G (IgG) was measured *via* indirect enzyme linked immunosorbent assay (ELISA). Briefly, 96-well Nunc MaxiSorp™ plates (Thermo Fisher Scientific) were coated with 250 ng/well of *B. canis* RM6/66 or *B. melitensis* 16M heat-killed sonicated lysate in coating buffer (pH 9.6, 0.05 M carbonate) at 4°C overnight. Plates were washed three times with PBST, and nonspecific binding was blocked with 200 μL of 3% skim milk in PBST at room temperature for 2 h. Following five washes, 2-fold dilutions of sera in PBST containing 1% skim milk were added and incubated at 37°C for 1 h. Plates were washed five times and HRP-labeled goat anti-mouse IgG (1:2000, KPL) was added, followed by incubation at 37°C for 1 h. Afterwards, OPD peroxidase substrate (Sigma-Aldrich) was added (100 μL/well) and incubated for 20 min at 37°C in the dark. The enzyme reaction was stopped by addition of 0.5M NaOH and absorbance was measured at 450 nm. Endpoint titers were reported as the log_10_ of the highest dilution giving an OD reading higher than 2 standard deviations above the mean of baseline sera. All assays were performed in triplicate, and the results are presented as the mean reciprocal endpoint titer.

### Preparation of single cell suspensions and *in vitro* stimulation

Spleens and lungs were collected from uninfected and infected mice at necropsy. To obtain single cell suspensions, spleens and lungs were processed by mechanical disruption using the Miltenyi Tissue Dissociator (Miltenyi Biotec) with the addition of a 30-min incubation at 37°C and gentle rotation for enzymatic digestion of the lungs using a Lung Dissociation kit (Miltenyi Biotec). Cell suspensions were filtered through a 70-µm cell strainer (VWR) and washed with PBS + 0.5% bovine serum albumin + 2 mM EDTA (PBSA) at 300 g for 8 min at 4°C. Red blood cells were lysed by addition of 3-4 mL of freshly diluted 1x RBC lysis buffer (eBioscience) for 3 min. After an additional wash in PBSA, cell suspensions were resuspended in complete media, filtered through a 40-µm cell strainer (VWR), and enumerated using a hemocytometer and trypan blue staining. Complete media was composed of RPMI-1640 (Gibco) with addition of 10% heat-inactivated fetal bovine serum (Equitech Bio), 10 mM sodium pyruvate (Gibco), 1x antibiotic/antimycotic (Gibco), and 50 µM 2-mercaptoethanol (Sigma).

For flow cytometric analysis of intracellular cytokine production, cells were aliquoted at 1x10^6^ cells/well in 200 µL of complete media into a 96-well U-bottom tissue culture plate (Falcon) and stimulated for 13 h with 25 µg of a heat-killed, sonicated lysate of *B. canis, B. abortus*, or *B. melitensis* followed by 4 h stimulation with 1x Protein Transport Inhibitor cocktail containing brefeldin A (eBioscience) to block cytokine secretion.

### Flow cytometry staining

Following overnight stimulation, cells were washed with PBS and stained with LIVE/DEAD Fixable Aqua dead cell stain (Thermo Fisher) for 30 min in the dark at 4°C to label dead cells. Nonspecific binding to the FcR was blocked by incubation with saturating doses of rat anti-mouse CD16/CD32 (BD Biosciences) for 15 min in the dark at 4°C. Afterwards, cells were labeled with antibodies specific for surface markers, including CD3 (clone 145-2C11, PE-eFluor 610), CD4 (clone GK1.5, eFluor 450), CD45 (clone 30-F11, APC-Cy7), and CD8 (clone 53.6.7, APC). Cells were fixed with IC Fixation Buffer (eBioscience), permeabilized with Permeabilization Buffer (eBioscience), and labeled with antibodies for intracellular markers, including IFN-γ (clone XMG1.2, FITC), TNF-α (clone MP6-XT22, PE), and IL-2 (clone JES6-5H4, PE-Cy7). All antibodies were obtained from eBioscience (CD3, CD4, IFN-γ, TNF-α) or Biolegend (CD45, CD8, IL-2) and were used at the manufacturers’ recommended dilutions. Fluorescence was acquired on a MoFlo Astrios cell sorter (Beckman Coulter) using the UltraComp eBeads (Thermo Fisher) and ArC Amine Reactive Compensation Beads (Thermo Fisher) to provide compensation controls. Additional controls included fluorescence minus one (FMO) controls and unstained cells. Results were analyzed using FlowJo software (Tree Star).

### Cytokine assays

For measurement of cytokines (IFN-ƴ, TNF-α, IL-10) in mouse organ homogenate supernatants, ELISA MAX Standard Sets (Biolegend) were used according to the manufacturer’s instructions.

The levels of TNF-α, IFN-ƴ, IL-2, IL-4, IL-10, IL-12p70, and IL-17A in culture supernatants from spleen and lung cells of infected mice were analyzed following culture of 10^6^ cells/well in 24-well plates for 48 hours with stimulation with *Brucella* spp. lysate or media using a Bio-Plex assay, according to the manufacturer’s instructions (Bio-Rad).

### Statistical analysis

Analysis was performed using GraphPad Prism software, version 6.0, San Diego, CA. The CFU data were normalized by log transformation and evaluated by two-way analysis of variance (ANOVA) repeated-measures test. Sidak’s multiple comparisons test was used to generate P values for mean comparisons. Splenic weights and endpoint antibody titers between groups and time points were analyzed using two-way ANOVA and P values were generated using Sidak’s multiple comparisons test or Tukey’s multiple comparisons tests, respectively. The percentage of histiocytic inflammation and levels of individual cytokines in examined organs were compared using one-way ANOVA and Tukey’s multiple comparisons test was used to generate P values. The percentage of T-lymphocyte subsets evaluated *via* flow cytometry were compared using two-way ANOVA and Sidak’s multiple comparisons test. In all analyses, a P value less than 0.05 constituted statistical significance.

## Results

### *Brucella canis* establishes a systemic infection following intratracheal inoculation in mice similar to smooth strains

While i.p. inoculation with *Brucella* spp. remains the most common approach to study the kinetics of *Brucella* spp, infection in mice, numerous studies in recent years have characterized the course of infection by smooth *Brucella* spp. in this model using the more natural mucosal route, including oral, intranasal, intratracheal, and whole-body aerosol inoculation (22, 34–38). However, *B. canis* infection has not been examined in mice following mucosal inoculation. To address this deficiency and to compare bacterial colonization and tissue tropism between rough *B. canis* vs its smooth counterparts, we inoculated mice intratracheally with 10^7^ CFU of *B. abortus* 2308*, B. melitensis* 16M, or *B. canis* RM6/66. Previous work in our laboratory demonstrated the need for a higher dose of 10^7^ CFU for *B. canis* to achieve the persistent systemic infection and the classic histologic lesions of infection seen with the typical 10^5^ CFU dose used with smooth *Brucella* spp (18). Some mice were also inoculated with a high dose of *B. canis*, 10^9^ CFU, as only this dose results in splenomegaly comparable to smooth strains following i.p. inoculation (18).

We utilized the PennCentury™ MicroSprayer because it allows for targeted delivery of a known inoculation dose directly to the lower airways through generation of microparticles with a mean size of 8 µm (32). Microparticles travel by centripetal force through the trachea and into the lower airways with deposition of particles on mucosal surfaces based on size, as occurs during respiration (39). Intratracheal inoculation with 10^7^ CFU of *B. canis* using this device demonstrated even distribution of the bacterium throughout all lung lobes (**Supplemental Figure 1**). Recent studies have utilized either intranasal or intratracheal inoculation, although it should be noted that intratracheal inoculation bypasses the nasal-associated lymphoid tissue and therefore comparison of immune responses between studies using different routes should be viewed with some caution (21, 37, 38).

**Figure 1:**
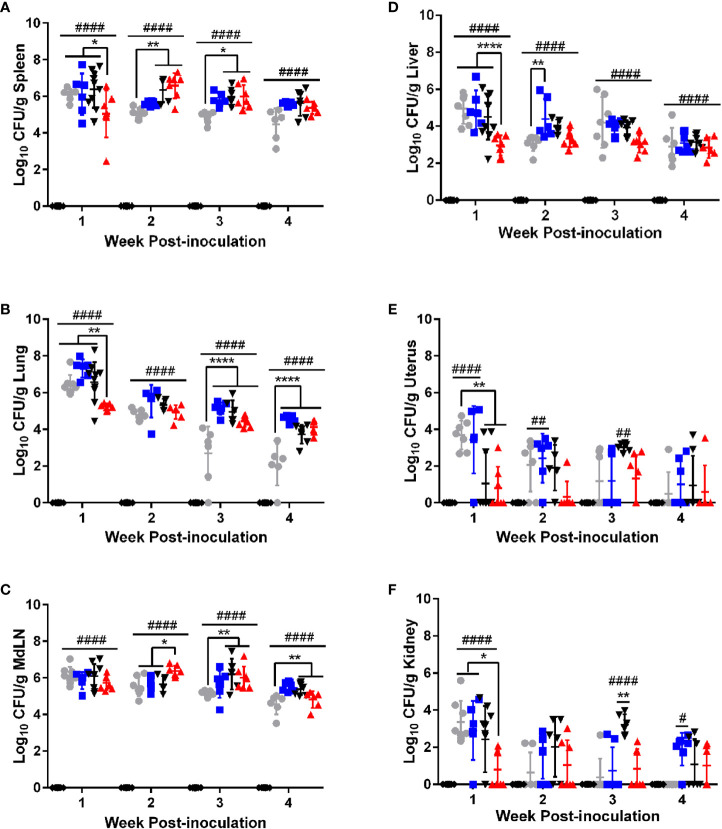
*B*. *canis* establishes persistent, systemic colonization following intratracheal inoculation comparable to smooth strains. Colonization from 1-4-weeks following inoculation of female C57BL/6J mice with 10^7^ CFU *B*. *melitensis, B. abortus*, or *B*. *canis*, 10^9^ CFU *B*. *canis*, or PBS in the **(A)** spleen, **(B)** lungs, **(C)** mediastinal lymph node (MdLN), **(D)** liver, **(E)** uterus, and **(F)** kidney. Data points represent the mean bacterial recovery in CFU per gram of tissue plus the standard deviation for all animals in each infection group at each time point. Data were analyzed using two-way ANOVA with Sidak’s multiple comparisons test. Significant differences are depicted between infection groups (*p < 0.05, **p < 0.01, ***p < 0.001, ****p < 0.0001) and between infection groups and the PBS negative control group (##p < 0.01, ####p < 0.0001).

Colonization was evaluated in target organs of *Brucella* infection: liver, spleen, and uterus. The lungs and mediastinal lymph node were also evaluated as these represent the first sites to be colonized following respiratory inoculation by smooth *Brucella* spp. with spread of bacteria from the lungs to the mediastinal lymph occurring within days of infection (19, 20). Finally, the kidney was included to gain an understanding of bacteremic spread as this tissue is not a target organ for *Brucella* spp. infection. Interestingly, during the first week post-infection, mice inoculated with 10^7^ CFU of *B. canis* exhibited significantly lower colonization in the spleen, lung, liver, uterus, and kidney compared to the smooth strains and the 10^9^ dose of *B. canis* (**Figure 1**). However, this difference was transient and starting from 2-4 weeks post-infection, *B. canis* colonized all target organs at similar levels compared to *B. abortus* or *B. melitensis* (**Figure 1**). Interestingly, *B. canis* showed higher colonization than *B. melitensis* in the spleen, lungs, and mediastinal lymph node at later time points (**Figures 1B–D**). Additionally, while differences were not significant, *B. canis* colonization increased in the spleen, liver, and mediastinal lymph node between 1- and 2-weeks post-infection, suggesting it there may be a delay in achieving the plateau phase of colonization compared to the smooth strains (**Figures 1A, C, D**).

Apart from the initial differences during 1-week post-infection, *B. canis* did not exhibit differences in colonization in mice inoculated with 10^9^ vs 10^7^ CFU (**Figure 1**). Thus, studies examining chronic timepoints of infection proceeded with 10^7^ CFU inoculation dose only (**Supplemental Figure 2**). Like the smooth strains, *B. canis* established a persistent infection in the spleen and mediastinal lymph node but slowly declined in the liver and lungs, although CFU were still detectable through 9-weeks post-infection in both organs (**Supplemental Figure 2A, C**). Colonization for all strains from 2-weeks post-infection onwards was highest in the well-known target organ, the spleen, but also the mediastinal lymph node (**Figures 1A, C**). Unexpectedly, *B. canis* persisted within the lungs at the same level as *B. abortus* and CFU was significantly higher than *B. melitensis*, which was unable to persist within the lungs and was cleared by 9-weeks post-inoculation (**Supplemental Figure 2B**).

### *B. canis* induces less severe inflammatory changes in target organs

**Figure 2:**
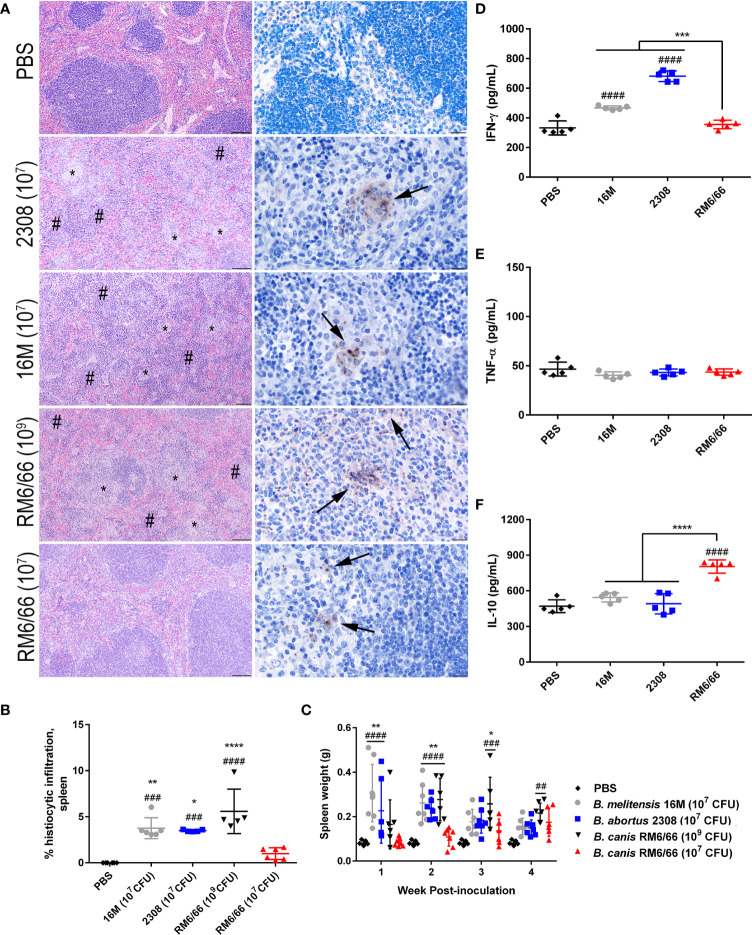
Intratracheal inoculation of mice with *B*. *canis* at 10^7^ CFU results in less splenic histiocytic inflammation than *B*. *abortus* or *B*. *melitensis* at the same dose and does not result in splenomegaly. **(A)** Representative histologic images of the spleen from each group at 2-weeks post-infection showing foci of histiocytic infiltration (*) and extramedullary hematopoiesis (#) in the left column (H&E stain, 10x magnification, scale bar= 100 µm) and positive intracytoplasmic immunolabeling for *Brucella* antigen (arrows) within these areas in the right column (IHC with DAB chromagen, 40x magnification, scale bar= 20 µm). **(B)** Quantification of % histiocytic infiltration within the spleen at 2-weeks post-infection. Data were analyzed *via* one-way ANOVA and Tukey’s multiple comparisons test. **(C)** Splenic weight was measured at 1-4 weeks post-inoculation and compared at each time point using two-way ANOVA and Sidak’s multiple comparisons test. Measurement of cytokines in spleen homogenate supernatants at 2-weeks post-infection, including **(D)** IFN-γ, **(E)** TNF-α, and **(F)** IL-10. Data were analyzed *via* one-way ANOVA and Tukey’s multiple comparisons test. Significant differences from *B*. *canis* 10^7^ CFU are depicted (**p < 0.01, ***p < 0.001, ****p < 0.0001) and between infection groups and the PBS negative control group (##p < 0.01, ####p < 0.0001).

Previous studies using i.p. inoculation in mice have indicated that *B. canis* appears to be attenuated compared to the smooth strains not only in terms of the dose required to achieve persistent infection but also in severity of histologic lesions (17, 18). At a dose of 10^7^ CFU following intratracheal inoculation, *B. canis* nevertheless exhibited comparable levels of colonization in all examined organs. We examined spleen, liver, and lung histologically at the peak of lesion severity, 2-weeks post-infection. Interestingly, we noted that despite similar colonization levels, *B. canis* induced significantly less granulomatous inflammation in the spleen than *B. melitensis* or *B. abortus* at the same dose (**Figure 2A**). This was observed despite similar levels of colonization and abundant *Brucella* antigen noted within the spleen *via* immunohistochemistry (**Figure 2A**, right column). Interestingly, only mice that received a high dose of 10^9^ CFU demonstrated significant granulomatous inflammation comparable to that induced by the smooth strains (**Figures 2A, B**). Quantification of the splenic lesions revealed that only *B. canis* at a dose of 10^9^ CFU resulted in significantly higher histiocytic infiltration than negative controls, as was observed when mice were inoculated with 10^7^ CFU *B. abortus* or *B melitensis* (**Figure 2B**). *B. canis* at 10^7^ CFU, on the other hand, did not result in significant histiocytic infiltration of the spleen, similar to negative control mice which received only PBS (**Figure 2B**).

A hallmark of *Brucella* infection in both mice and natural host species is splenomegaly. Interestingly, only *B. canis* at a high dose of 10^9^ CFU was able to induce significant splenomegaly, at a level comparable to that induced by the smooth strains (**Figure 2C**). *B. canis* at a dose of 10^7^ CFU, however, produced no detectable change in spleen size throughout the course of infection. The increase in spleen size in mice inoculated with *B. abortus, B. melitensis*, and the high dose of *B. canis* corresponded to marked extramedullary hematopoiesis (EMH) which widely separated lymphoid follicles (**Figure 2A**, left column, #). To determine if these stark differences in histologic inflammation corresponded to differences in pro- and anti-inflammatory cytokines, spleen homogenate supernatants were analyzed for levels of IFN-γ, TNF-α, and IL-10. Corresponding to more severe inflammatory lesions and larger spleen size, infection with *B. melitensis* or *B. abortus* induced significant IFN-γ in the spleen while infection with 10^7^ CFU *B. canis* did not (**Figure 2D**). In contrast, only infection with *B. canis* induced significant production of IL-10 in the spleen (**Figure 2F**). As with the spleen, TNF-α in the liver supernatants did not differ between groups.

Similar differences were also noted in the liver, with *B. abortus, B. melitensis*, and the high dose of *B. canis* inducing numerous granulomas while *B. canis* at 10^7^ CFU produced only scattered granulomas (**Supplemental Figure 3A**). Quantification of these lesions revealed the same trend as in the spleen, with only *B. abortus, B. melitensis*, and the high dose of *B. canis* inducing significantly higher histiocytic infiltration than the PBS negative control group (**Supplemental Figure 3B**). Unlike in the spleen, however, no significant differences in IFN-γ or IL-10 in liver homogenate supernatants were noted between groups (**Supplemental Figures 3C–E**). As with the spleen TNF-α did not differ between groups.

**Figure 3:**
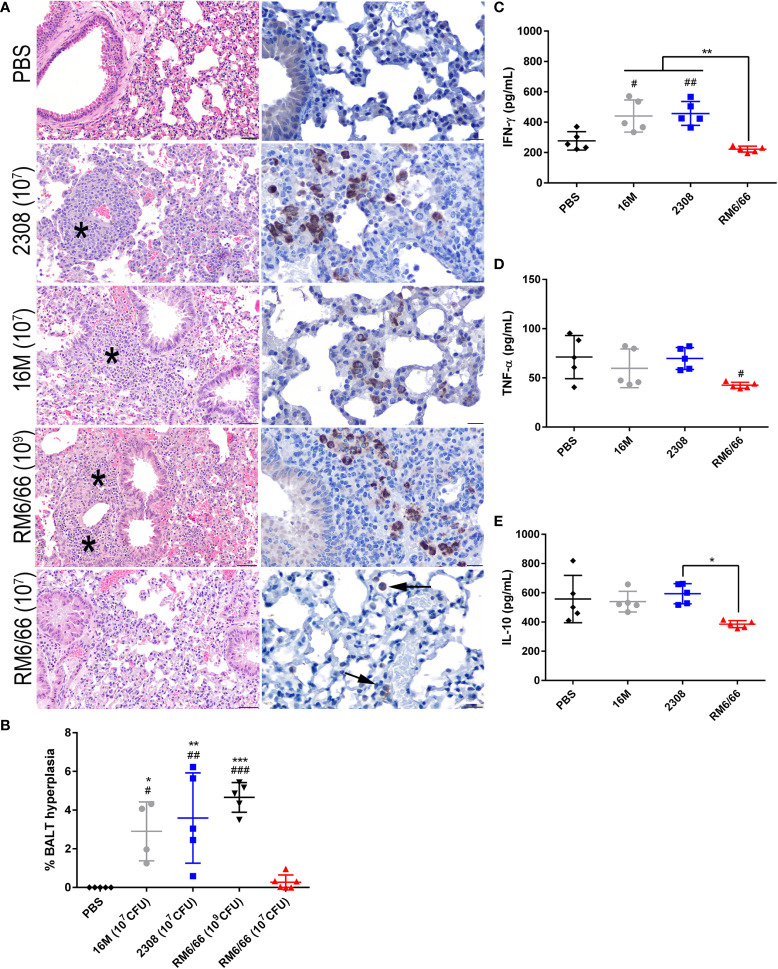
Intratracheal inoculation with *B*. *abortus* or *B melitensis* results in BALT hyperplasia in the lungs while *B*. *canis* only induces this change at a higher dose. **(A)** Representative histologic images of the lungs at 20x (left column, H&E stain, scale bar= 50 µm) from each group at 2-weeks post-infection showing thick accumulations of lymphocytes with fewer plasma cells and macrophages surrounding bronchioles (BALT hyperplasia, indicated by *) in mice inoculated with *B*. *abortus, B. melitensis*, or a high dose of *B*. *canis*. *Brucella* antigen was detected within the cytoplasm of macrophages by immunohistochemistry in infected animals (right column, IHC with DAB chromagen, 40x magnification, scale bar= 20 µm). **(B)** Quantification of % BALT hyperplasia within the lung at 2-weeks post-inoculation. Measurement of cytokines in lung homogenate supernatants at 2-weeks post-infection, including **(C)** IFN-γ, **(D)** TNF-α, and **(E)** IL-10. Data were analyzed using *via* one-way ANOVA and Tukey’s multiple comparisons test. Significant differences from *B*. *canis* 10^7^ CFU are depicted (*p < 0.05, **p < 0.01, ***p < 0.001) and between infection groups and the PBS negative control group (#p < 0.05, ##p < 0.01, ###p < 0.001).

Previous studies using aerosol or intranasal inoculation of mice by smooth strains of *Brucella* have not noted any significant histologic lesions in the lungs, although these typically utilize a dose of 10^4^-10^6^ CFU (36, 38, 40). The mice of this study inoculated with 10^7^ CFU *B. abortus* or *B. melitensis* or 10^9^ CFU *B. canis* developed significant hyperplasia of bronchial associated lymphoid tissue (BALT). These changes consisted of thick rims of lymphocytes with fewer macrophages surrounding bronchioles and adjacent blood vessels (**Figure 3A**). The lungs of mice in all infection groups contained numerous alveolar macrophages filled with abundant *Brucella* antigen identified immunohistochemically, both adjacent to areas of BALT hyperplasia and throughout the alveoli (**Figure 3A**, right panel). BALT hyperplasia was absent in the PBS negative control group and in mice inoculated with 10^7^ CFU *B. canis*. Following quantification of percentage of tissue taken up by BALT hyperplasia, all infection groups except for those inoculated with 10^7^ CFU *B. canis* demonstrated a significant increase over PBS negative controls (**Figure 3B**). As noted in the spleen, only inoculation with *B. melitensis* or *B. abortus* resulted in significant induction of IFN-γ in the lung homogenate supernatants (**Figure 3C**). TNF-α induced by *B. canis* was lower compared to controls while IL-10 production in *B. canis*-infected mice was lower compared to B. abortus in contrast to that seen in the spleen (**Figures 3D, E**).

### Intratracheal inoculation of *B. canis* results in a stronger humoral immune response

**Figure 4:**
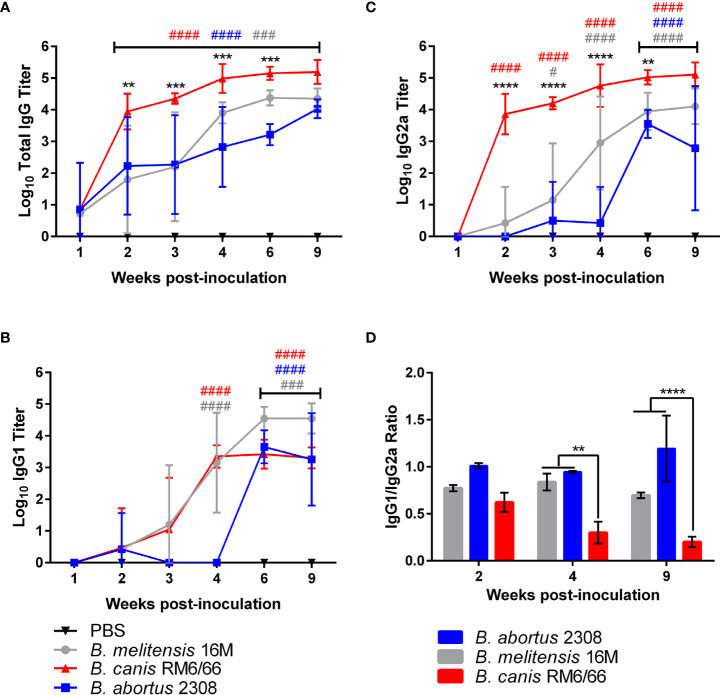
*Brucella canis* induces a stronger total anti-*Brucella* IgG response with a greater skew towards IgG2a. *Brucella*-specific humoral immune response in mice inoculated intratracheally with *B*. *canis, B. abortus*, or *B*. *melitensis*. Sera was collected from weeks 1-4, 6- and 9-weeks post-inoculation for measurement of total IgG **(A)**, IgG1 **(B)**, and IgG2a **(C)**. Mean titers were compared between groups at each time point using two-way ANOVA and Tukey’s multiple comparisons test. Significant differences are depicted between *B*. *canis* and both *B*. *abortus* and *B*. *melitensis* (**p < 0.01, ***p < 0.001, ****p < 0.001) and between each infection group and the PBS negative control group (##p < 0.01, ###p < 0.001, ####p < 0.0001). **(D)** The ratio of IgG1:IgG2a was compared across infection groups. Significant differences are depicted between infection groups (**p < 0.01, ***p < 0.001, ****p < 0.001).

After noting the differences in severity of inflammation in target organs despite similar levels of colonization, we next assessed for differences in the humoral immune response between rough *B. canis* and smooth *B. abortus* and *B. melitensis*. As expected, all strains induced a significant *Brucella*-specific total IgG response beginning at 2-weeks post-inoculation and lasting throughout the study (**Figure 4A**). However, *B. canis* induced significantly higher total IgG titers from 2- to 6-weeks post-infection. Dissection of this response revealed that while titers of *Brucella*-specific IgG1 did not differ between strains (**Figure 4B**), *B. canis* induced significantly higher IgG2a titers from 2- to 6-weeks post-infection (**Figure 4C**).

When comparing the ratio of IgG1 to IgG2a, *B. canis* was shown to induce a stronger skew towards IgG2a at 4- and 9-weeks post-infection compared to *B. abortus* and *B. melitensis*, corresponding to the higher titers of *Brucella*-specific IgG2a (**Figure 4D**). A ratio favored more towards IgG2a in mice is typically associated with a skew towards the more protective Th1, or cell-mediated immune response. Interestingly, these differences in IgG1:IgG2a ratios did not reflect differences in tissue colonization between the strains.

### Previous infection protects mice against secondary *B. canis* challenge

**Figure 5:**
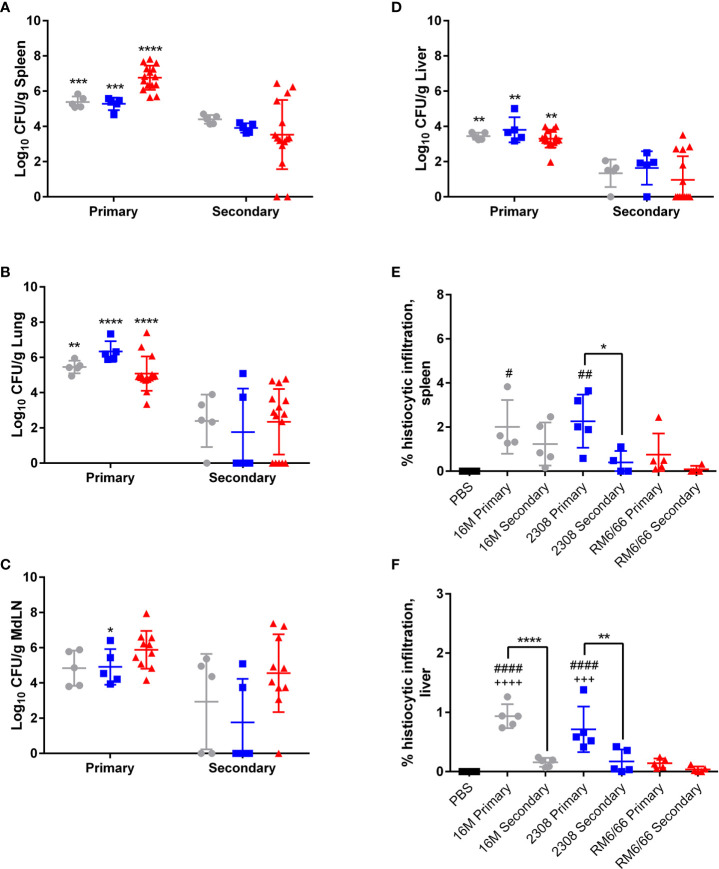
Previous infection protects mice against secondary intratracheal challenge with *B*. *canis, B. abortus*, or *B*. *melitensis.* Colonization following inoculation of female C57BL/6J mice with 10^7^ CFU *B*. *melitensis, B abortus*, or *B*. *canis* in the **(A)** spleen, **(B)** lungs, **(C)** mediastinal lymph node (MdLN), and **(D)** liver. Data points represent the mean bacterial recovery in CFU per gram of tissue plus the standard deviation for all animals in each infection group 2-weeks following primary infection or secondary challenge. Data were analyzed using two-way ANOVA with Sidak’s multiple comparisons test. Quantification of % histiocytic infiltration within the **(E)** spleen and **(F)** liver at 2-weeks post-infection. Data were analyzed *via* one-way ANOVA and Tukey’s multiple comparisons test. Significant differences are depicted between infection groups (*p < 0.05, **p < 0.01, ***p < 0.001, ****p < 0.0001) and between infection groups and the PBS negative control group (##p < 0.01, ####p < 0.0001).

After determining that *B. canis* induces a milder inflammatory response in mice during primary infection, we next sought to analyze the impact of LPS phenotype on the protective immune response. Additionally, while the immune response to smooth *Brucella* spp. has been extensively investigated, far less is known regarding the protective response against *B. canis.* Analysis of the memory immune response is commonly performed following challenge in vaccinated mice. However, a model of secondary infection with the wild-type organism represents an alternative approach which has recently begun to be explored with smooth *Brucella* spp (20, 21, 28, 29). In such a model, mice are infected with *Brucella* spp., rested for 4 weeks to allow for a memory immune response to develop, treated with antibiotics to clear the primary infection, and finally reinfected with the same organism to analyze the induced memory response. Mice were inoculated intratracheally with wild-type *B. canis, B. abortus*, or *B. melitensis* and then treated with antibiotics to clear the infection. After a rest period and confirmation that mice had cleared the infection through sacrifice of a subset of mice from each group, mice were challenged with the same infectious organism and euthanized at 2-weeks post-infection. Notably, previous exposure to *B. canis, B. abortus*, and *B. melitensis* provided significant protection against colonization following secondary challenge in the spleen, liver, and lung as well as overall decreased colonization in the mediastinal lymph node (**Figures 5A–D**). It is important to note, however, that although protection was significant, sterile immunity was not achieved in any organ following challenge with any *Brucella* strain.

Previous infection also reduced the severity of histiocytic inflammation in the spleen and liver following challenge, although this was only significant for the smooth strains in the liver and for *B. abortus* alone in the spleen (**Figures 5E, F**). Significant protection in the spleen or liver was not offered by previous infection with *B. canis*, although primary infection resulted in near-negligible inflammation in these organs (**Figures 5E, F**).

### T lymphocyte responses during primary and secondary infection differs between rough and smooth *Brucella* spp.

We next investigated if the memory immune response against *B. canis* differed from its smooth counterparts following secondary challenge, a key step towards rational vaccine design. To do this, IFN-γ, TNF-α, and IL-2 production by T lymphocyte populations was analyzed both systemically (spleen) and locally (lungs) following primary infection and secondary challenge with the homologous strain *via* flow cytometry. It is crucial to evaluate both the local and systemic responses following mucosal challenge as previous work with *B. melitensis* in mice has shown differences in the time course and nature of the memory immune response in the lungs vs the spleen (20). The gating strategy is outlined in **Supplemental Figure 4**.

**Figure 6:**
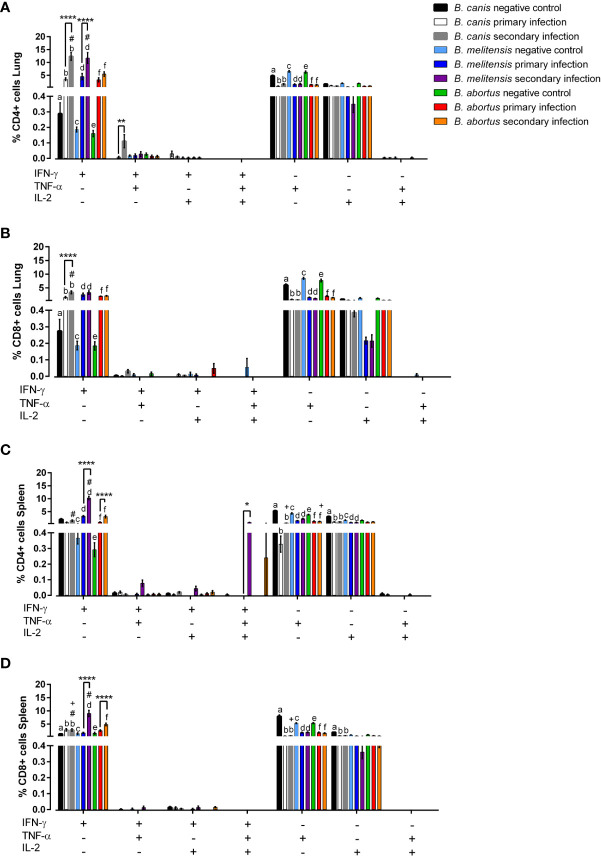
Rough and smooth *Brucella* spp. induce significant IFN-γ-producing T-lymphocytes, more so during secondary exposure, but only smooth *Brucella* do so at the systemic level. Flow cytometric analysis of T-lymphocyte cytokine production was performed following primary or secondary inoculation of female C57BL/6J mice with 10^7^ CFU *B*. *melitensis, B. abortus*, or *B*. *canis*. **(A)** CD4+ and **(B)** CD8+ T-lymphocytes were evaluated in the lungs and **(C)** CD4+ and **(D)** CD8+ T lymphocytes were also evaluated in the spleen. Data were analyzed using two-way ANOVA with Sidak’s multiple comparisons test. Significant differences between groups are indicated (**p < 0.01, ***p< 0.001, ****p < 0.0001), between groups and the *B*. *abortus* secondary infection group (#p < 0.05), and between groups and the *B*. *melitensis* secondary infection group (+p < 0.05). There are significant differences (p < 0.05) in the graph between “a” and “b,” “c” and “d,” and “e” and “f.

Locally within the lungs, both primary and secondary infection with all examined *Brucella* spp. resulted in significant production of IFN-γ by CD8+ and CD4+ T lymphocytes (**Figures 8A, B**). Interestingly, IFN-γ production by CD4+ T lymphocytes in the lungs was higher following secondary challenge compared to primary infection with the homologous strain for all examined *Brucella* spp., although this change was only significant for *B. canis* and *B. melitensis* (**Figure 6A**).

As in the lungs, significant induction of IFN-γ by CD4+ and CD8+ T lymphocytes was noted in the spleens of mice during secondary infection with *B. abortus* or *B. melitensis* (**Figure 6D**). IFN-γ also significantly increased following secondary challenge compared to primary infection for mice infected with smooth *Brucella* spp. Interestingly, however, neither primary nor secondary infection with *B. canis* induced IFN-γ by CD4+ T lymphocytes in the spleen (**Figure 6C**).

Unexpectedly, TNF-α production by CD4+ and CD8+ T lymphocytes in both the lungs and spleen of uninfected control mice was significantly higher compared to mice infected with any of the examined *Brucella* spp. and there were no significant differences between primary and secondary infection in any group (**Figure 6**). None of the *Brucella* spp. induced significant IL-2 production the lungs or spleen during primary or secondary infection. In fact, IL-2 production was occasionally lower in infected than uninfected mice (**Figures 6C, D**). Due to the low percentages of cells producing TNF-α or IL-2 in any group, it is unclear if these changes represent downregulation triggered by *Brucella* spp. infection.

Apart from significant IFN-γ induction, polyfunctionality was not notably induced by any *Brucella* spp., neither during primary nor secondary infection. Secondary infection with *B. canis* resulted in significantly higher induction of CD4+ T lymphocytes in the lungs producing both IFN-γ and TNF-α (**Figure 6A**) while significant induction of trifunctional CD4+ T lymphocytes was noted during secondary infection with *B. melitensis* in the spleen (**Figure 6C**). Importantly, polyfunctional T lymphocytes were otherwise not associated with *Brucella* spp. infection despite their association with protective immunity against other intracellular pathogens (41, 42).

### Primary and secondary infection with smooth *Brucella* spp. stimulates stronger Th1-biased cytokine production

**Figure 7:**
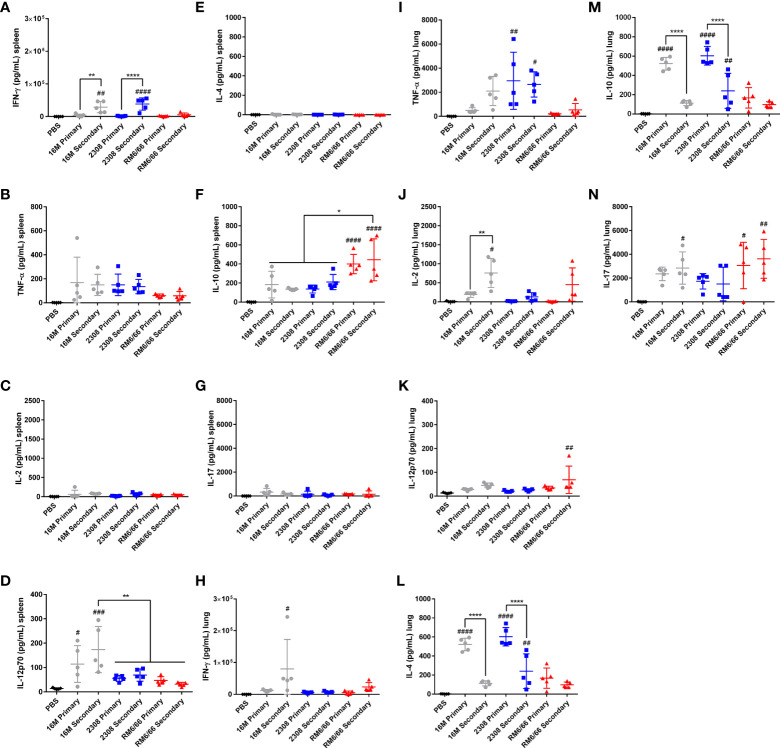
Secondary infection with *B*. *abortus* or *B*. *melitensis*, but not *B*. *canis*, results in a Th1 skewed cytokine response in the spleen and lungs. Measurement of cytokines in splenocyte culture supernatant after 48-hour stimulation with *Brucella* lysate from mice during primary or secondary infection with *Brucella* spp. **(A)** IFN-γ, **(B)** TNF-α, **(C)** IL-2, **(D)** IL-12p70, **(E)** IL-4, **(F)** IL-10, and **(G)** IL-17. Measurement of cytokines in lung cell culture supernatant after 48-hour stimulation with *Brucella* lysate from mice during primary or secondary infection with *Brucella* spp. **(H)** IFN-γ, **(I)** TNF-α, **(J)** IL-2, **(K)** IL-12p70, **(L)** IL-4, **(M)** IL-10, and **(N)** IL-17. Data were analyzed using *via* one-way ANOVA and Tukey’s multiple comparisons test. Significant differences are indicated from the PBS negative control group (#p < 0.05, ##p < 0.01, ###p < 0.001, ####p < 0.0001) or between groups (*p < 0.05, **p < 0.01, ****p < 0.0001).

To assess if the differences numbers of cytokine-producing T lymphocytes coincided with functional differences that could account for differences in degree of histologic inflammation, cytokine levels following primary or secondary challenge were also evaluated in cell culture supernatant using Bio-Plex technology in the spleen and lungs (**Figure 7**). Mirroring the results seen *via* flow cytometry, significant IFN-γ production in the spleen was observed only with smooth *Brucella* spp. secondary infection. Interestingly, both primary and secondary infection with *B. canis* was associated with significant IL-10 production in the spleen while this change was not observed for the smooth strains (**Figures 7A, F**).

In the lungs, both IL-4 and IL-10 were significantly induced following infection with smooth *Brucella* spp., but levels were significantly lower with secondary compared to primary infection (**Figures 7L, M**). Classic Th1 cytokines were induced during secondary infection with smooth *Brucella* spp. in the lungs, including IFN-γ with *B. melitensis* and TNF-α with *B. abortus* (**Figures 7H, I**). This Th1 skew to the memory immune response in the lungs was only observed with the smooth *Brucella* spp. and not with *B. canis*, with which all cytokine levels apart from IL-17 remained low (**Figure 7N**).

### Previous infection provides superior protection over vaccination against *B. canis* challenge in mice

**Figure 8:**
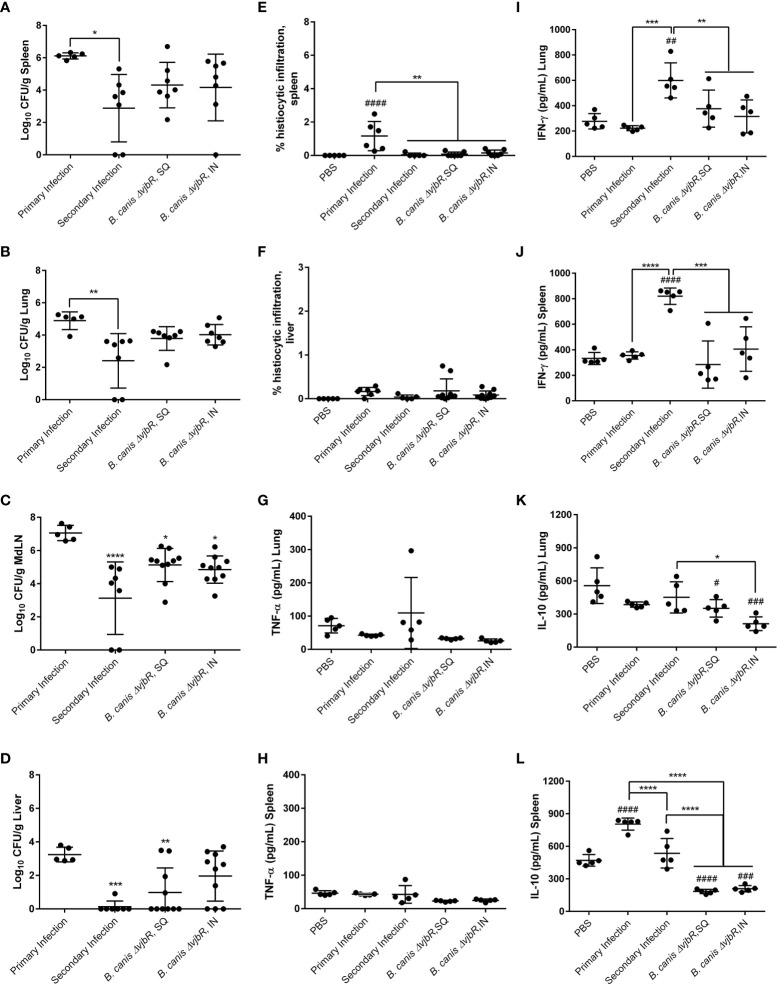
Previous infection or vaccination, regardless of route, protect mice against secondary intratracheal challenge with *B*. *canis* but superior protection is offered by previous infection, associated with higher IFN-γ and IL-10 compared to vaccination. Mice were infected with 10^7^ CFU *B*. *canis* or vaccinated with 10^9^ CFU of *B*. *canis ΔvjbR* subcutaneously or intranasally. Vaccinated mice were challenged at 6-weeks post-vaccination. Colonization 2-weeks post-primary infection or challenge of female C57BL/6J mice with 10^7^ CFU*B. canis* in the **(A)** spleen, **(B)** lungs, **(C)** mediastinal lymph node (MdLN), and **(D)** liver. Data points represent the mean bacterial recovery in CFU per gram of tissue plus the standard deviation for all animals in each infection group. Quantification of % histiocytic infiltration within the **(E)** spleen and **(F)** liver at 2-weeks post-infection. Measurement of cytokines in lung and spleen homogenate supernatants at 2-weeks post-challenge, including **(G, H)** TNF-α, **(I, J)** IFN-γ and **(K, L)** IL-10. Data were analyzed using *via* one-way ANOVA and Tukey’s multiple comparisons test. Data were analyzed *via* one-way ANOVA and Tukey’s multiple comparisons test. Significant differences are depicted between infection groups (*p < 0.05, **p < 0.01, ***p < 0.001, ****p < 0.0001) and between infection groups and the PBS negative control group (#p < 0.05, ##p < 0.01, ###p < 0.001, ####p < 0.0001).

To compare the memory immune response associated with previous infection with *B. canis* to that induced by vaccination, we vaccinated mice with a live attenuated vaccine (LAV) candidate, *B. canis ΔvjbR*, using both a parenteral (subcutaneous) and mucosal (intranasal) route. Despite a previous study showing significant protection against colonization following i.p. challenge in vaccinated mice, only mice that had previously been infected with wild-type *B. canis* showed significant protection against colonization both systemically (spleen, liver) and locally (lungs, mediastinal lymph node) following secondary intratracheal challenge (**Figures 8A–D**) (31). In contrast, mice that were vaccinated, regardless of route, did not show significant protection at the systemic level or locally within the lungs (**Figures 8A–B, D**). Rather, protection was only observed within the mediastinal lymph node in vaccinated mice (**Figure 8C**). Interestingly, the route of vaccination did not impact protection on the local level, with no significant differences in colonization noted post-challenge in the lungs or mediastinal lymph node between vaccinated groups (**Figures 8B, C**). However, despite modest reduction in colonization following challenge afforded by vaccination, both previous infection and vaccination, regardless of route, protected against histiocytic inflammation in the spleen, a key pathological feature of *Brucella* spp. infection (**Figure 8E**). This effect was not observed in the liver, however, although little inflammation was detected even during primary infection (**Figure 8F**).

To compare bacterial colonization and tissue pathology with production of pro- and anti-inflammatory cytokines, we measured the levels of TNF-α, IFN-γ, and IL-10 in organ homogenate supernatants. No significant differences in TNF-α were noted between groups (**Figures 8G, H**). Interestingly, however, significantly higher levels of IFN-γ were noted in mice that had previously been infected with *B. canis* compared to mice that had been vaccinated in both the lungs and spleen (**Figures 8I, J**). In contrast, vaccination but not previous infection resulted in significantly lower levels of IL-10 in the lungs and spleen following challenge (**Figures 8K, L**).

The induction of IFN-γ in the spleen following secondary challenge in mice previously infected with *B. canis* and the drop in IL-10 in both lung and spleen differs from the flow cytometry results. However, cytokine levels in organ homogenate supernatants encompasses production by cells types other than CD4+ or CD8+ T lymphocytes, including macrophages.

## Discussion

Brucellosis is predominantly contracted across mucosal surfaces with strong evidence that the protective immune response to *Brucella* spp. differs between infection routes in mice (20, 21). This emphasizes the need for animal models utilizing a physiologically relevant inoculation route to investigate immune correlates and understand the required immune components associated with the particular vaccine being tested. *Brucella canis*, distinct from the other zoonotic species with its rough LPS, has not previously been evaluated following mucosal infection in mice and the nature of a protective immune response against this organism has remained little explored. With vaccination still representing the most effective tool to combat brucellosis, we sought to contribute towards vaccine development against this pathogen by establishing a mouse model of mucosal *B. canis* infection and using it to compare components of the protective memory response against *B. canis* RM6/66 vs. its smooth counterparts, *B. abortus* 2308 and *B. melitensis* 16M (6, 10–14).

Following inoculation *via* the respiratory route in mice, lung colonization typically rises during the first few days to weeks and then gradually declines while bacteria are reach the liver and spleen by 1-2 weeks (34, 37, 38, 43, 44). In this study, rough *B. canis* was able to colonize all organs at the same level as smooth *B. abortus* and *B. melitensis*. Interestingly, *B. melitensis* was cleared from the lungs by 9-weeks post-infection, while *B. canis* and *B. abortus* plateaued, indicating that this organ may serve as a persistence niche for these strains. Previous studies have also noted the inability of *B. melitensis* to persist within the lungs, in contrast to *B. abortus* (36, 37). Smooth *Brucella* spp. are known to traffic from the lungs to the draining mediastinal lymph nodes *via* alveolar macrophages within days of infection, a trend which was noted for smooth and rough *Brucella* in this study (45). Notably, all three strains sustained high levels of colonization in the lymph nodes at levels as high as the spleen, the persistence niche most frequently evaluated in brucellosis studies in mice. It is thus advisable that draining lymph nodes be evaluated in vaccine efficacy studies as colonization following challenge may be missed if only the spleen is evaluated.

Despite similar colonization levels, *B. canis* produced milder inflammatory lesions in the spleen and liver compared to *B. abortus* and *B. melitensis*. Interestingly, this corresponded to increased IFN-γ, a known Th1 proinflammatory cytokine, in spleen and lung supernatants for the smooth strains and increased IL-10, an anti-inflammatory cytokine, in spleen supernatants for *B. canis*. Chacón-Díaz etal. (17) noted that *B. canis* induced fewer hepatic granulomas and significantly lower IFN-γ in the serum than *B. abortus* (17). This work and ours support the hypothesis that *B. canis* causes less inflammation in multiple organs compared to smooth *B. abortus* and *B. melitensis*, although it should be noted that *B. canis* can induce significant inflammation in its natural host and a laboratory animal model may not recapitulate all findings noted during infection in the natural host (5). In contrast to the liver or spleen, lesions in the lungs have seldom been reported with *Brucella* infection in mice. However, we noted significant BALT hyperplasia in mice infected with smooth *Brucella* spp., presumably due to the higher dose of 10^7^ CFU utilized in this study. BALT hyperplasia represents a nonspecific, reactive proliferation of immune cells in the lungs in response to infection or inflammation (46). Notably, at the same inoculation dose, *B. canis* induced no BALT hyperplasia. Whether BALT induction is harmful or beneficial to the host is context dependent and may exacerbate pulmonary inflammation or contribute to adaptive immunity against pathogens (46–48). As no differences in lung colonization were observed during the peak of BALT formation in this study, any functional significance during *Brucella* infection is uncertain.

*B. canis* also exhibited differences from its smooth counterparts during the humoral immune response. Despite the same inoculation, *B. canis* significantly higher IgG2a titers and a stronger skew towards this IgG subtype, indicative of a Th1-biased immune response (16). Interestingly, this did not correspond to any differences in colonization but did coincide with decreased inflammatory lesions. Although higher IgG2a titers are typically used as a marker of protective efficacy in brucellosis vaccine studies, increased production of specific IgG2a has been shown to benefit *B. abortus* by increasing opsonization and thereby enhancing macrophage invasion (49). Therefore, higher specific IgG2a might be advantageous to *B. canis* and allow it to establish wider macrophage infection compared to smooth strains, although this remains to be examined.

A central goal of characterizing mucosal *B. canis* infection in mice is to serve as a model for investigating the protective immune response against this pathogen, both in the context of natural infection and vaccination. We utilized the format of secondary infection, recently elaborated for *B. melitensis* by Muraille, E. and colleagues to begin this endeavor (20, 21). As has been shown for smooth *Brucella* spp., previous infection with *B. canis* provided widespread protection against colonization and tissue pathology in multiple organs. However, this protection was not significant within the draining mediastinal lymph node in mice infected with either smooth or rough *Brucella* spp., again indicating that this location could serve as a persistence niche that should be included in vaccine efficacy studies. Sterile protection was not noted in most mice as in previous studies which may be related to the higher dose and earlier time point of our study, although it is also possible that achieving sterile immunity in all animals may not be feasible with *Brucella* infection (20, 21).

The secondary infection model was used to begin exploring factors involved in the protective immune response. Our results confirm previous work indicating the key importance of a strong Th1 response centered on T-lymphocytes producing IFN-γ, long known for smooth *Brucella* in the context of i.p. infection and recently confirmed for respiratory infection (20, 21). Indeed, a dominant Th2 immune response has been shown to favor smooth *Brucella* growth in the lungs and IFN-γ produced by either CD4+ or CD8+ T-lymphocytes is crucial for control following nasal infection in mice (20, 21). Such a Th1-biased immune response was observed in our study in mice infected with smooth *B. abortus* and *B. melitensis* through strong IFN-γ induction in both lungs and spleen. However, it appears that rough *B. canis* not only incites a weaker Th1 response at the systemic level with lower IFN-γ production in the spleen, but also shifts the immune response towards a more anti-inflammatory profile through induction of IL-10. These trends in cytokine profiles may explain the reduced inflammatory lesions in *B. canis-*infected mice and deserve further exploration. It would also be intriguing to extend these studies to assess for differences in the protective memory immune response in mice previously infected by smooth *Brucella* spp. and challenged with rough *B. canis*, and vice versa.

While most previous studies into the immune response to brucellosis focused on monofunctional T-lymphocytes, polyfunctionality is being increasingly investigated in response to other intracellular pathogens (41, 50–54). Such work has shown that CD4+ T-lymphocytes secreting only IFN-γ have a limited capacity to develop into memory cells compared with those also producing IL-2 and IFN-γ may synergize with TNF-α to mediate killing of intracellular pathogens (41, 42). Interestingly, neither smooth nor rough *Brucella* infection in this study resulted in widespread induction of polyfunctional T-lymphocytes. Such results have been noted previously with *Campylobacter jejuni*, in which secondary infection did not result in significant induction of polyfunctional T-lymphocytes, with possible explanations including a low induction of memory T-lymphocytes or development of T-lymphocyte exhaustion (55, 56). Exhaustion has been demonstrated in mice chronically infected with smooth *Brucella*, in which CD8+ T-lymphocytes could transition into IFN-γ-producing memory cells but with a lack of polyfunctional activity (57). Neither smooth nor rough *Brucella* spp. induced significant TNF-α production during primary or secondary infection, with TNF-α being higher in negative controls. It may be that *Brucella* spp. are suppressing TNF-α production, as has been noted to occur under the influence of its virulence factors including Omp25 and TcpB (58, 59). Our results indicate little polyfunctional activity induced by *Brucella* infection, regardless of LPS phenotype, further evidence that *Brucella* suppresses the immune response to favor its persistence. Additional studies at later time points are required to determine if T-lymphocyte exhaustion is also occurring in this context.

Finally, we compared protective efficacy provided by previous infection with *B. canis* to that afforded by vaccination with our previously developed LAV, *B. canis ΔvjbR*. Although significant protection against colonization was afforded by previous infection and not by to vaccination, vaccinated mice were protected against development of inflammatory lesions in the spleen. As sterile immunity was not noted even following previous infection, this may not represent the most feasible outcome for a brucellosis vaccine and reduction in tissue pathology with resultant disease may be a more important and achievable endpoint. While significant protection has been noted following i.p. challenge in mice vaccinated with *B. canis*, *ΔvjbR*, previous studies have not evaluated challenge *via* the respiratory route. This difference in route may account for the lack of vaccine-induced protection against colonization in this study. Additionally, as no reference vaccine exists for *B. canis*, it is possible that the model of secondary infection could serve as a replacement when assessing vaccine candidates in mice.

The superior protection afforded by previous exposure over vaccination coincided with a greater induction of IFN-γ in the spleen and lungs. This suggests that vaccines against *B. canis* should be designed to induce this cytokine. Additionally, while greater levels of IL-10 were also noted in previously infected mice following challenge compared to vaccinated mice, a significant drop in IL-10 was noted in the spleen between primary and secondary infection. This trend was also noted in IL-10 production by T lymphocytes *via* flow cytometry for smooth *Brucella* spp. It appears likely that a drop in IL-10 production following challenge is associated with superior protection for both smooth and rough *Brucella* spp. and deserves further exploration.

*Brucella canis* differs from smooth *B. abortus, B. melitensis*, and *B. suis* not only in its rough LPS phenotype, but also in the lack of a protective vaccine and a limited understanding on immune correlates against this pathogen. We have developed a mouse model for mucosal *B. canis* infection to address these knowledge gaps, with the understanding that route of infection significantly impacts the required protective immune response. Despite similar levels of colonization, *B. canis* induced a less robust inflammatory response in examined organs, both at the histologic level during primary infection and systemically during the memory response, coinciding with a more even balance between Th1 and Th2 cytokines and increased production of IL-10. These results suggest that *B. canis* is even more effective at evading immune detection and destruction than smooth *B. abortus* and *B. melitensis*, with the ability to persist in the absence of a strong inflammatory immune response. *B. canis*, like the smooth strains, also induced little polyfunctional T cell activity locally and systemically, suggesting the ability to suppress the memory immune response in *Brucella* spp. is independent of LPS phenotype.

## Data availability statement

The original contributions presented in the study are included in the article/Supplementary Material. Further inquiries can be directed to the corresponding author.

## Ethics statement

This study was reviewed and approved by Institute of Animal Care and Use Committee at Texas A&M University.

## Author contributions

AA-G and LS designed the study. LS, DG-G, and MH performed the experiments and acquired the data. LS analyzed the data and wrote the manuscript. AA-G revised the original draft. All authors contributed to the article and approved the submitted version.

## Funding

Student stipend support was provided by the National Institutes of Health Institutional Training Grant T32 fellowship 5 OD 11083-10 (LS, MH). Research support was provided by the National Institutes of Health International Research Scientist Development Award (IRSDA/K01) K01 TW00998-1 (AA-G).

## Conflict of interest

The authors declare that the research was conducted in the absence of any commercial or financial relationships that could be construed as a potential conflict of interest.

## Publisher’s note

All claims expressed in this article are solely those of the authors and do not necessarily represent those of their affiliated organizations, or those of the publisher, the editors and the reviewers. Any product that may be evaluated in this article, or claim that may be made by its manufacturer, is not guaranteed or endorsed by the publisher.

## Supplementary material

Supplementary Figure 1 Distribution of *Brucella canis* following intratracheal inoculation in mice is even throughout the lungs. Mice were anestheized and inoculated intratracheally using the PennCentury™ MicroSprayer IA-1C with 10^7^ CFU of *B. canis.* The lungs were divided into 4 quadrants with the right cranial quadrant containing the right superior and middle lobes and the right caudal quadrant containing the right inferior and post-caval lobes. Data points represent the mean bacterial recovery in CFU per mL plus the standard deviation. Data were analyzed *via* one-way ANOVA and Tukey’s multiple comparisons test.

Supplementary Figure 2
*B. canis* establishes a chronic infection following intratracheal inoculation comparable to *B. abortus* but with higher colonization than *B. melitensis* in the lungs. Colonization 6 and 9 weeks following inoculation of female C57BL/6J mice with 10^7^ CFU *B. melitensis, B. abortus*, or *B. canis*, or PBS in the (A) spleen, (B) lungs, (C) mediastinal lymph node (MdLN), (D) liver, (E) uterus, and (F) kidney. Data points represent the mean bacterial recovery in CFU per gram of tissue plus the standard deviation for all animals in each infection group at each time point. Data were analyzed using two-way ANOVA with Sidak’s multiple comparisons test. Significant differences are depicted between infection groups (*p < 0.05, **p < 0.01, ***p < 0.001, ****p < 0.0001) and between infection groups and the PBS negative control group (##p < 0.01, ####p < 0.0001).

Supplementary Figure 3 Intratracheal inoculation of mice with *B. canis* at 10^7^ CFU results in less granulomatous inflammation in the liver than *B. abortus* or *B. melitensis* at the same dose. (A) Representative histologic images of the liver from each group at 2-weeks post-infection showing foci of histiocytic infiltration/granulomas (arrows) in the left column (H&E stain, 10x magnification, scale bar= 100 µm) and positive intracytoplasmic immunolabeling for *Brucella* antigen (arrows) within the granulomas in the right column (IHC with DAB chromagen, 40x magnification, scale bar= 20 µm). (B) Quantification of % histiocytic infiltration within the liver at 2-weeks post-inoculation. Measurement of cytokines in liver homogenate supernatants at 2-weeks post-infection, including (D) IFN-γ, (E) TNF-α, and (F) IL-10. Data were analyzed *via* one-way ANOVA and Tukey’s multiple comparisons test. Significant differences from *B. canis* 10^7^ CFU are depicted (**p < 0.01, ***p < 0.001, ****p < 0.0001) and between infection groups and the PBS negative control group (##p < 0.01, ####p < 0.0001).

Supplementary Figure 4 Gating strategy for evaluation of single or simultaneous production of TNF-α, IFN-γ, and IL-2 by CD4+ and CD8+ T-lymphocytes in mice following primary or secondary intratracheal inoculation with *Brucella* spp. Single cells were gated based on viability and T-lymphocytes were selected by double expression of CD45 and CD3. CD4+ and CD8+ populations were then separately analyzed or production of the three cytokines.
