# Supplementary figures and images for: Primary and memory immune responses against rough Brucella canis are less robust compared to smooth B. abortus and B. melitensis following intratracheal infection in mice
Source: Front Immunol. 2022 Aug 11;13:959328. doi: 10.3389/fimmu.2022.959328 (PMC9402402; doi:10.3389/fimmu.2022.959328)

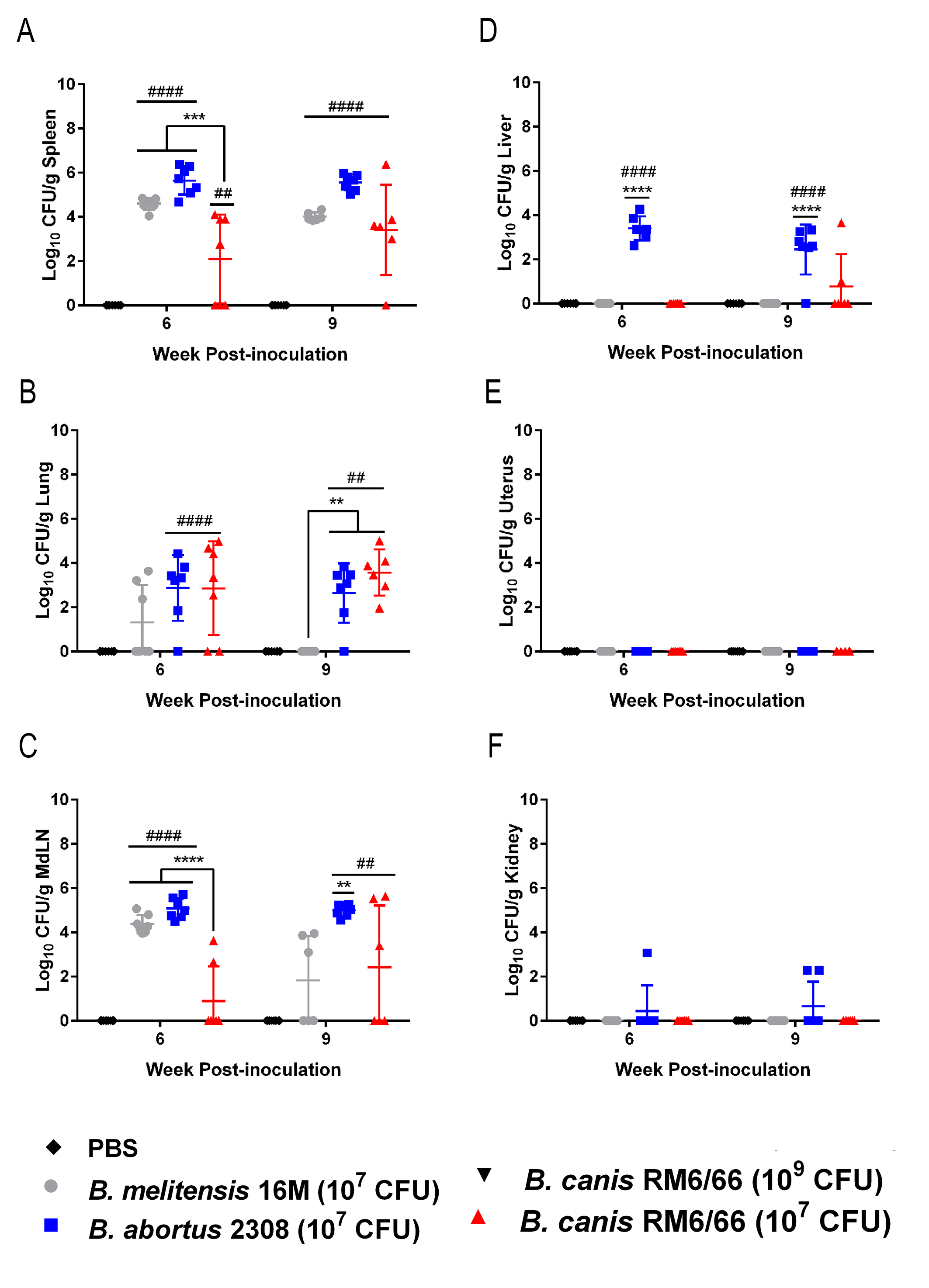

Supplement: Supplementary Figure 2 — B. canis establishes a chronic infection following intratracheal inoculation comparable to B. abortus but with higher colonization than B. melitensis in the lungs. Colonization 6 and 9 weeks following inoculation of female C57BL/6J mice with 107 CFU B. melitensis, B. abortus, or B. canis, or PBS in the (A) spleen, (B) lungs, (C) mediastinal lymph node (MdLN), (D) liver, (E) uterus, and (F) kidney. Data points represent the mean bacterial recovery in CFU per gram of tissue plus the standard deviation for all animals in each infection group at each time point. Data were analyzed using two-way ANOVA with Sidak’s multiple comparisons test. Significant differences are depicted between infection groups (*p < 0.05, **p < 0.01, ***p < 0.001, ****p < 0.0001) and between infection groups and the PBS negative control group (##p < 0.01, ####p < 0.0001). [file Image_2.tiff]

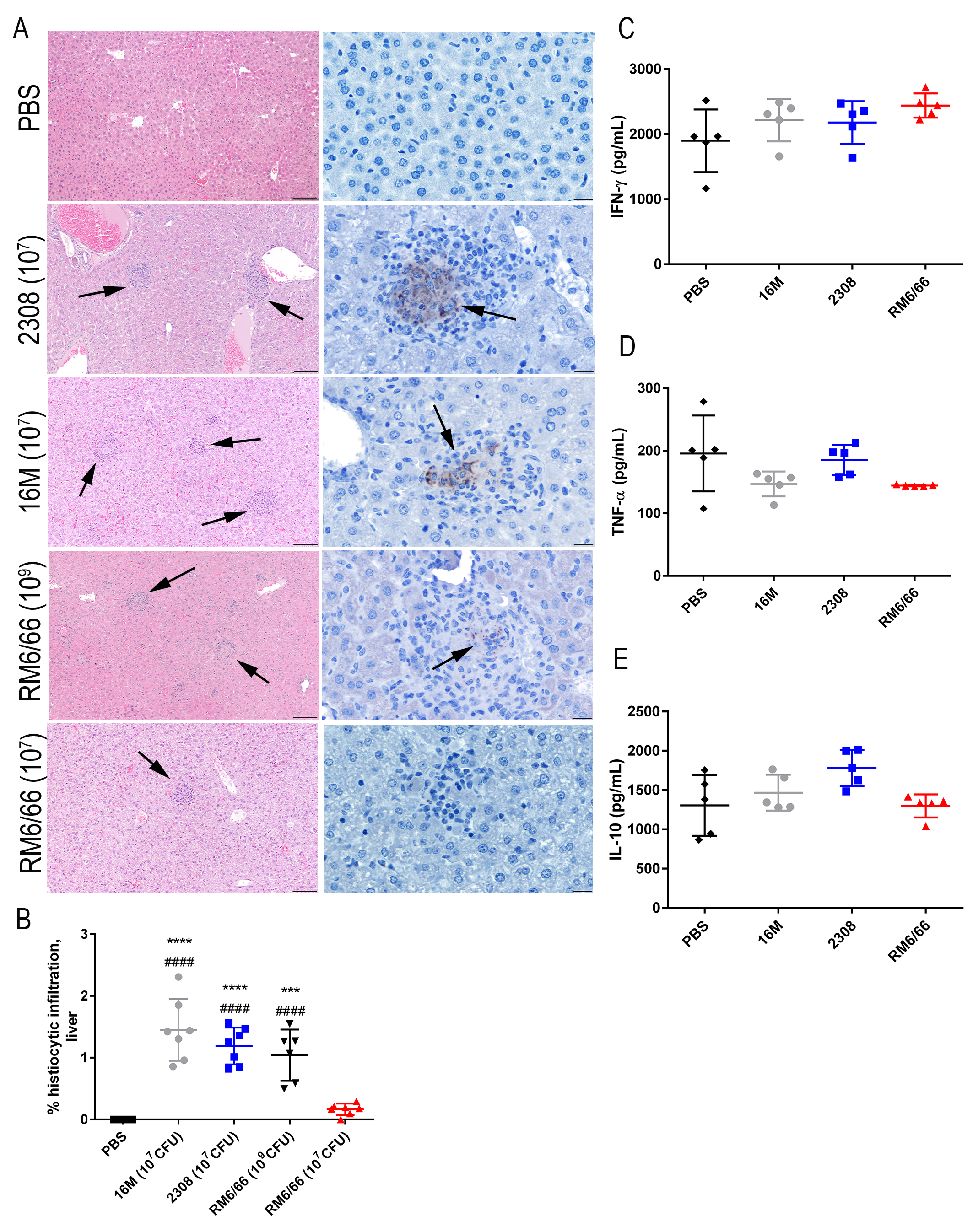

Supplement: Supplementary Figure 3 — Intratracheal inoculation of mice with B. canis at 107 CFU results in less granulomatous inflammation in the liver than B. abortus or B. melitensis at the same dose. (A) Representative histologic images of the liver from each group at 2-weeks post-infection showing foci of histiocytic infiltration/granulomas (arrows) in the left column (H&E stain, 10x magnification, scale bar= 100 µm) and positive intracytoplasmic immunolabeling for Brucella antigen (arrows) within the granulomas in the right column (IHC with DAB chromagen, 40x magnification, scale bar= 20 µm). (B) Quantification of % histiocytic infiltration within the liver at 2-weeks post-inoculation. Measurement of cytokines in liver homogenate supernatants at 2-weeks post-infection, including (D) IFN-γ, (E) TNF-α, and (F) IL-10. Data were analyzed via one-way ANOVA and Tukey’s multiple comparisons test. Significant differences from B. canis 107 CFU are depicted (**p < 0.01, ***p < 0.001, ****p < 0.0001) and between infection groups and the PBS negative control group (##p < 0.01, ####p < 0.0001). [file Image_3.tiff]

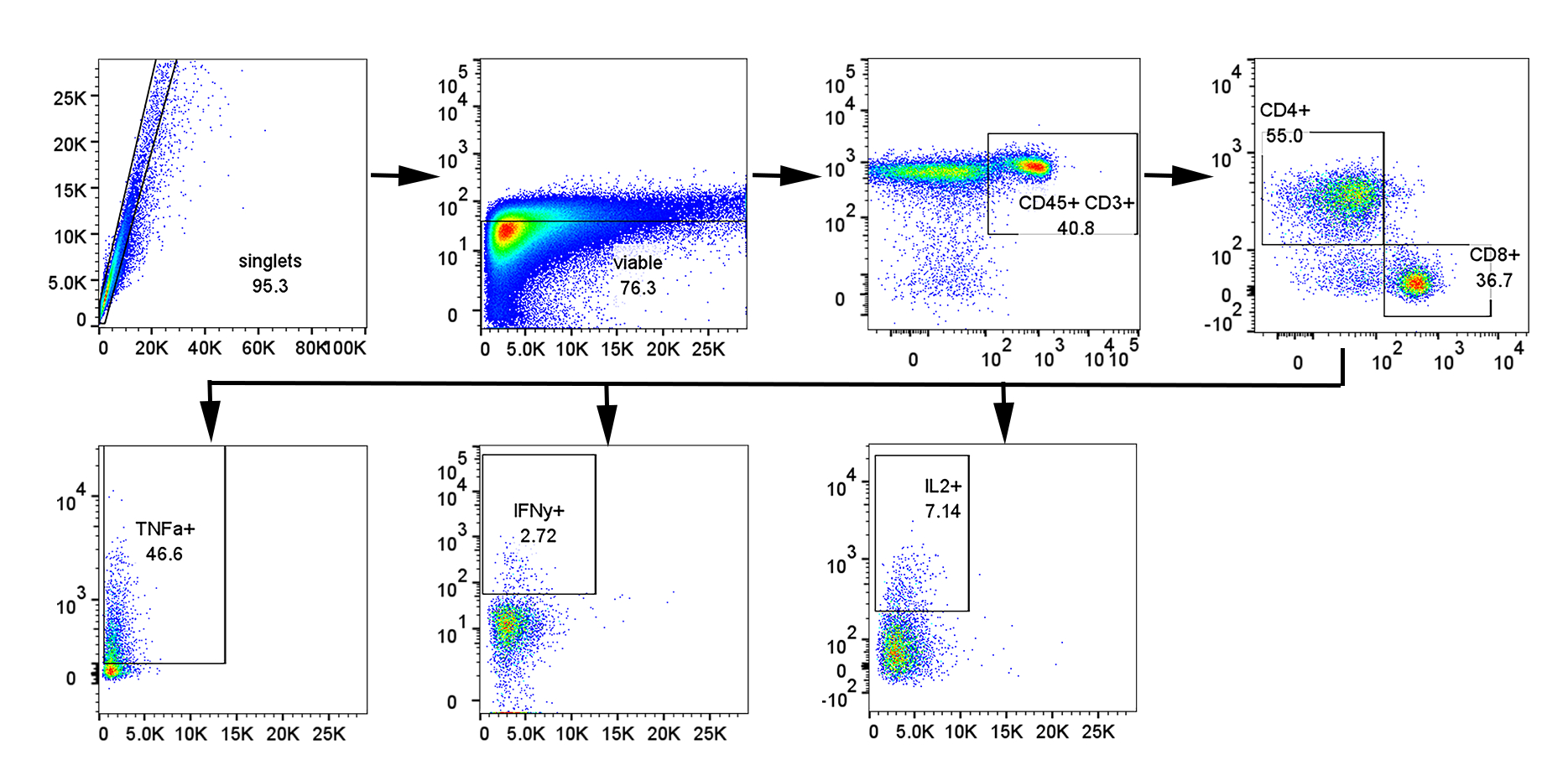

Supplement: Supplementary Figure 4 — Gating strategy for evaluation of single or simultaneous production of TNF-α, IFN-γ, and IL-2 by CD4+ and CD8+ T-lymphocytes in mice following primary or secondary intratracheal inoculation with Brucella spp. Single cells were gated based on viability and T-lymphocytes were selected by double expression of CD45 and CD3. CD4+ and CD8+ populations were then separately analyzed or production of the three cytokines. [file Image_4.tiff]
